# KIF11 and KIF15 mitotic kinesins are potential therapeutic vulnerabilities for malignant peripheral nerve sheath tumors

**DOI:** 10.1093/noajnl/vdz061

**Published:** 2020-01-04

**Authors:** Ernest Terribas, Marco Fernández, Helena Mazuelas, Juana Fernández-Rodríguez, Josep Biayna, Ignacio Blanco, Gabriela Bernal, Irma Ramos-Oliver, Craig Thomas, Rajiv Guha, Xiaohu Zhang, Bernat Gel, Cleofé Romagosa, Marc Ferrer, Conxi Lázaro, Eduard Serra

**Affiliations:** 1 Program of Predictive and Personalized Medicine of Cancer (PMPPC), Germans Trias & Pujol Research Institute (IGTP), Badalona, Barcelona, Spain; 2 Cytometry Core Facility, Germans Trias & Pujol Research Institute (IGTP), Badalona, Barcelona, Spain; 3 Hereditary Cancer Program, Catalan Institute of Oncology (ICO-IDIBELL-ONCOBELL), L’Hospitalet de Llobregat, Barcelona, Spain; 4 Clinical Genetics and Genetic Counseling Program, Germans Trias i Pujol Hospital, Barcelona, Spain; 5 Department of Pathology, Vall d’Hebron University Hospital, Barcelona, Spain; 6 National Center for Advancing Translational Sciences, National Institutes of Health, Chemical Genomics Center, Bethesda, Maryland, USA; 7 Centro de Investigación Biomédica en RED (CIBERONC), Instituto de Salud Carlos III, Madrid, Spain

**Keywords:** BRD4 inhibitor, combined treatment, KIF11 inhibitor, KIF15, malignant peripheral nerve sheath tumor (MPNST)

## Abstract

**Background:**

Malignant peripheral nerve sheath tumor (MPNST) constitutes the leading cause of neurofibromatosis type 1–related mortality. MPNSTs contain highly rearranged hyperploid genomes and exhibit a high division rate and aggressiveness. We have studied in vitro whether the mitotic kinesins KIF11, KIF15, and KIF23 have a functional role in maintaining MPNST cell survival and can represent potential therapeutic vulnerabilities.

**Methods:**

We studied the expression of kinesin mRNAs and proteins in tumors and cell lines and used several in vitro functional assays to analyze the impact of kinesin genetic suppression (KIF15, KIF23) and drug inhibition (KIF11) in MPNST cells. We also performed in vitro combined treatments targeting KIF11 together with other described MPNST targets.

**Results:**

The studied kinesins were overexpressed in MPNST samples. KIF15 and KIF23 were required for the survival of MPNST cell lines, which were also more sensitive than benign control fibroblasts to the KIF11 inhibitors ispinesib and ARRY-520. Co-targeting KIF11 and BRD4 with ARRY-520 and JQ1 reduced MPNST cell viability, synergistically killing a much higher proportion of MPNST cells than control fibroblasts. In addition, genetic suppression of *KIF15* conferred an increased sensitivity to KIF11 inhibitors alone or in combination with JQ1.

**Conclusions:**

The mitotic spindle kinesins KIF11 and KIF15 and the cytokinetic kinesin KIF23 play a clear role in maintaining MPNST cell survival and may represent potential therapeutic vulnerabilities. Although further in vivo evidences are still mandatory, we propose a simultaneous suppression of KIF11, KIF15, and BRD4 as a potential therapy for MPNSTs.

**Key Points**

1. MPNST cell lines are more sensitive to KIF11 inhibitors than nontumoral fibroblasts.
2. Co-targeting KIF11/BRD4 shows synergistic antitumoral effects, especially in KIF15-deficient MPNST cells.
3. A triple KIF11–KIF15–BRD4 inhibition may represent a potential therapy for MPNSTs.

**Importance of the Study**

MPNSTs are aggressive tumors with hyperploid genomes. There is a lack of efficient treatments for MPNSTs which constitute the leading cause of NF1-related mortality. In this work, we describe that mitotic kinesins, the molecular motors that travel along microtubules during cell division, are highly expressed in MPNSTs. In vitro suppression of mitotic kinesins KIF11, KIF15, and KIF23 in MPNST cells greatly affects their viability and cell cycle progression. We compile in vitro data supporting a triple KIF11–KIF15–BRD4 inhibition as a potential therapeutic vulnerability for MPNSTs.

Neurofibromatosis type 1 (NF1) is a hereditary cancer syndrome caused by mutations in the *NF1* gene, which encodes neurofibromin, a negative regulator of Ras protein. NF1 presents with several and variable clinical manifestations that affect various tissues. The most distinctive trait is a high predisposition to develop several tumors, especially but not exclusively, tumors of the peripheral nervous system.^1^ Among them, dermal neurofibromas (DNFs) are the most frequent, affecting almost all (~99%) NF1 patients. Around 50% of NF1 patients have plexiform neurofibromas (PNFs), which originate from multiple nervous fascicles.^2^ Some PNFs transform into a type of soft tissue sarcoma called malignant peripheral nerve sheath tumor (MPNST). From PNF or independently, a distinct nodular lesion can appear, characterized by increased cellularity and the presence of atypia. These atypical neurofibromas are considered premalignant lesions and from which an MPNST may end progressing, or not.^3^ Around 50% of MPNSTs are associated with NF1 patients, while the other half develop sporadically.^4^ These are aggressive tumors, with an invasive growth, propensity to metastasize, and limited sensitivity to chemotherapy and radiation. So far, surgical resection is the basis of its clinical management. MPNST has a poor prognosis and it is the leading cause of NF1-related mortality.^5^

MPNSTs contain highly rearranged hyperploid genomes characterized by the occurrence of many genomic alterations and a low point mutation burden.^6^ Some of these alterations include known tumor suppressor genes and oncogenes driving MPNST pathogenesis. Recurrent mutations in NF1-associated MPNSTs, in addition to *NF1* loss, involve the deletion of the *CDKN2A*/*B* locus^3^ and the inactivation of components of the polycomb repressive complex 2 (PRC2) *SUZ12* and *EED*.^6,7^

Several potential therapeutic targets have been described in preclinical studies. These are mainly involved in the Ras signaling pathway and other cellular processes, such as angiogenesis, apoptosis, epigenetic regulation, and mitosis,^8^ and some of them have reached studies in clinical trials for MPNSTs with single agents, but with no conclusive results after phase II. Given this limited success, recent and ongoing clinical trials for the treatment of MPNSTs include several combined therapies.^9^

Kinesins are proteins that act as molecular motors traveling unidirectionally along microtubules in a cell. The kinesin superfamily is represented by 45 genes in humans. They have 2 main roles in cell physiology: transport of intracellular vesicles and organelles^10^ and cell division.^11^ At present, 16 kinesins have been involved in participating at different stages of mitosis and cytokinesis and most of them have been found deregulated in several cancer types.^12^ Mitotic kinesins represent mitosis-specific targets that bypass the neurotoxicity produced by other antimitotic drugs impairing microtubule polymerization, hence some kinesins have emerged as potential targets for the development of antitumoral drugs.^13^

So far, the role of mitotic kinesins in MPNST pathogenesis has not been studied. As MPNSTs are aggressive and hyperploid tumors, exhibiting a high proliferation rate and bearing many chromosomes, we wondered whether mitotic kinesins may be important for MPNST survival. We used several in vitro functional assays to explore their role in MPNST cells and provided evidence to suggest they may be potential therapeutic targets for MPNSTs. We also searched for synergistic and efficient combined treatments including some kinesin members and other MPNST targets, as a first in vitro preclinical study to be considered for further in vivo preclinical studies.

## Materials and Methods

### Cell Lines and Primary Cultures

Several cell lines derived from MPNSTs were used: S462 and ST88-14 (provided by Dr Nancy Ratner), sNF96.2 (provided by Dr Thomas De Raedt), 90-8 and the sporadic MPNST-derived STS-26T (provided by Dr Eric Legius), and sporadic HS-Sch-2 (obtained from RIKEN). In addition, the commercial human foreskin fibroblast (HFF) cell line CCD-1112Sk (ATCC) was used as a normal control cell line. All cells were cultured with supplemented high glucose Dulbecco’s Modified Eagle Medium (DMEM) under standard conditions (see Supplementary Extended Methods). NF1-associated MPNST cell lines were authenticated using the *NF1* described mutation and we performed an Short Tandem Repeat (STR) profile in all lines (Terribas et al., manuscript in preparation).

Schwann cell (SC) primary cultures obtained from 8 DNFs and 4 PNFs were used as control benign cells. They were obtained from NF1 patients who gave their informed consent and after Institutional Review Board (IRB) approval. SCs were isolated from these tumors and cultured as previously described^14^ (see Supplementary Extended Methods).

### Immunohistochemistry

A tissue microarray including 16 PNF and 14 MPNST samples (see Supplementary Extended Methods) was used to check the expression of KIF11 and KIF15. In addition, the proliferation marker Ki67 was also immunodetected. Formalin-Fixed Paraffin-Embedded (FFPE) tissue sections 3 μm thick were incubated with anti-KIF11 antibody (Proteintech), anti-KIF15 antibody (Proteintech), or Ki67 (Ventana Medical Systems), and a horseradish peroxidase–conjugated secondary antibody was used in all cases. Immunohistochemical staining was performed on the Ventana Benchmark XT Automated IHC Stainer (Ventana Medical Systems Inc.; see Supplementary Extended Methods).

### Quantitative Reverse Transcription Polymerase Chain Reaction

RNA was extracted from cells using Tripure Isolation Reagent (Roche) and retrotranscribed using Superscript III reverse transcriptase (see Supplementary Extended Methods), and cDNA was submitted to qPCR in a Light-Cycler 480 Real-Time PCR System. The sequences of the primers and probes used and conditions for amplification can be found in Supplementary Extended Methods. A Microsoft Excel spreadsheet was used to analyze qPCR data for relative expression calculations as described^15^ (see Supplementary Extended Methods).

### Protein Extraction and Western Blot

Total protein was extracted after cell lysis with Radioimmunoprecipitation assay buffer (see Supplementary Extended Methods). An Sodium Dodecyl Sulfate-Polyacrylamide Gel Electrophoresis was performed using 20 µg of protein per sample. Polyvinylidene Fluoride membranes were incubated with either anti-KIF11 antibody (Proteintech) or anti-KIF15 antibody (Proteintech) at 4ºC overnight and with anti-α-tubulin (Sigma-Aldrich) during 1 h at room temperature. Membranes were then incubated with IRDye 680LT and IRDye 800CW secondary antibodies (LI-COR) for 1 h at room temperature and scanned using the Odyssey Imaging System (LI-COR; see Supplementary Extended Methods).

### siRNA Transfection

KIF15 and KIF23 mRNA expression was knocked down in both S462 and ST88-14 cell lines using siRNA molecules. A siRNA pool targeting KIF15, KIF23, or a non-targeting control (NTC; siGENOME SMARTpool, Dharmacon) was introduced into cells using lipofectamine RNAiMAX (Life Technologies). Transfected cells were used for RNA extraction and for several functional in vitro assays (see Supplementary Extended Methods).

### CRISPR/Cas Edition

We used CRISPR/Cas technique to edit the *KIF15* gene and generate a *KIF15*^KO^ S462 clone. For the generation of the CRISPR constructs, we selected the pX330-U6-Chimeric_BB-CBh-HSpCas0 (Addgene) and the pRGS2 surrogate reporter (Labomics) vectors (see Supplementary Extended Methods).

S462 cells derived from a single cell clone were transfected with both pX330 and pRGS2 constructs with Lipofectamine 2000 (Life Technologies), and sorted, after 3 days, using the BD FACSAria II. Single Red Fluorescent Protein-positive and Green Fluorescent-positive sorted cells were plated in 96-well plates, cultured, and expanded in standard conditions (see Supplementary Extended Methods). A viable S462 clone was found to be KIF15-deficient (Supplementary Figure S1) and was used together with KIF15^WT^ S462 clonal cells for single and combined treatments.

### In Vitro Single Drug Treatment

HFFs, ST88-14, and S462 were incubated in a dose-dependent manner with either ispinesib (Selleckchem) or ARRY-520 (MedChemExpress), which selectively inhibit KIF11.

In ispinesib treatment, for the dose–response time course, cells were seeded in 96-well plates and treated with 4 concentrations, and cell viability was measured at 0 h, 24 h, and 48 h. For the calculation of the half-maximal inhibitory concentration (IC_50_), cells were plated in a 6-well plate and treated with 4 concentrations. After 48 h, cells were counted with the Countess Automated Cell Counter (Life Technologies). For the dose–response of ispinesib treatment after *KIF15* depletion, we combined siRNA transfection of *KIF15* and the NTC siRNAs (Dharmacon), and chemical inhibition of KIF11 with ispinesib in S462 cell line. Here, S462 cells were plated in a 10 cm plate and transfected with siRNA pools (targeting *KIF15* or an NTC) and lipofectamine RNAiMAX (Life Technologies). After 24 h, cells were counted and plated on a 6-well plate. After 24 h, cells were treated with 4 concentrations of ispinesib and after 48 h were counted with the Countess Automated Cell Counter (Life Technologies; see Supplementary Extended Methods)

In ARRY-520 treatment, for both the dose–response time course and the IC_50_ calculation, cells were seeded in 96-well plates, treated with 4 concentrations of ARRY-520. Cell viability was measured at 0 h, 24 h, 48 h, and 72 h in the time course and at 72 h for IC_50_ calculation (see Supplementary Extended Methods).

### Immunofluorescence of α-Tubulin

The effect of the KIF11 inhibitors ispinesib and ARRY-520 in the microtubule cytoskeleton was determined by immunofluorescence of α-tubulin. In short, MPNST cells were plated on an 8-well Labtek slide (Sarstedt) and incubated with DMSO, 2.5 nM ispinesib or 1 μM ARRY-520 for 24 h. Cells were then incubated with 1:100 of anti-α-tubulin-Alexa488 antibody (Life Technologies) for 45 min. Cells were then mounted with Vectashield with DAPI (Vector Laboratories) and visualized at 100× in a fluorescence microscope (Leica DMI 6000B).

### Mid-Throughput Combined Treatment Screening

The MPNST cell lines S462 and sNF96.2 were screened in a 10 × 10 matrix combination format with 500 cells per 5 μl well with ispinesib or ARRY-520 in combination with other 20 compounds from the MIPE 4.0 library of approved and investigational drugs, to assess synergy. Matrix blocks were dispensed using an acoustic dispenser (EDC Biosystems), and 48-h CellTiter-Glo readout was used to inform on cell viability.

### In Vitro Combined Drug Treatment

We performed a dose–response experiment where HFFs, ST88-14, and S462 were treated with 6 concentrations of single ARRY-520, 6 concentrations of the single second drug, and 6 combined concentrations of ARRY-520 and the second drug, in a time course. Cell viability was measured at 0 h, 24 h, 48 h, and 72 h of treatment (see Supplementary Extended Methods). We also studied the effect of the ARRY-520/JQ1 combination in the viability, proliferation, cell cycle, and apoptosis of HFFs, ST88-14, S462, and S462-*KIF15*^KO^ (see Supplementary Extended Methods).

### Cell Viability Analysis

Cell viability was assessed using several methodologies (XTT assay, RealTime-Glo MT assay, and automated cell counting) depending on the performed experiment.

For the siRNA-transfected ST88-14 cells, viability was measured using the XTT assay (Roche; see Supplementary Extended Methods). For the siRNA-transfected S462 cell line, viability was assessed by automated cell counting with the Countess Automated Cell Counter (Life Technologies; see Supplementary Extended Methods).

In the in vitro ispinesib treatment, for the dose–response time course, cell viability was measured with XTT assay; and for the IC_50_ determination, automated cell counting was performed (see Supplementary Extended Methods). In the in vitro ARRY-520 treatment, for both the dose–response time course and the IC_50_ calculation, the RealTime-Glo MT assay was used (see Supplementary Extended Methods). This assay was also used for assessing the viability of the combined drug treatments.

### Cell Proliferation and Cell Cycle Analysis

The Click-iT EdU Alexa Fluor 488 Flow Cytometry Assay Kit (Life Technologies) was used in combination with propidium iodide (PI, Sigma) staining for the calculation of proliferating cells and cell cycle phases using the flow cytometer in siRNA transfection experiments and in combined drug treatments (see Supplementary Extended Methods). A BD LSRFortessa SORP cytometer was used to measure the amount of Alexa Fluor 488 dye (EdU-positive cells) and PI (DNA content).

### Apoptosis Analysis

Apoptosis was measured in siRNA transfection experiments and in combined drug treatments using the Annexin-V-Alexa Fluor 568 antibody (Roche and Life Technologies). This was combined with bis-benzimide dye (Sigma) staining for the detection of late apoptotic and necrotic cells (see Supplementary Extended Methods). A BD LSRFortessa SORP cytometer was used to measure the amount of Alexa Fluor 568 dye (Annexin-V-positive apoptotic cells) and bis-benzimide (late apoptotic/necrotic cells).

### Anchorage-Independent Growth Analysis

Soft agar assay was performed on the S462 cell line to assess their anchorage-independent growing properties after siRNA delivery or during drug treatment. ST88-14 cell line does not form colonies in vitro. In 6-well plates, an S462 cell suspension was mixed with agarose, plated, and allowed to solidify. Plates were incubated under standard conditions for 2 weeks. Colonies were fixed and stained with crystal violet for 1 h, and plates were scanned (see Supplementary Extended Methods).

## Results

### Mitotic Kinesins KIF11, KIF15, and KIF23 Are Highly Expressed in MPNSTs and Derived Cell Lines

**Figure 1.**
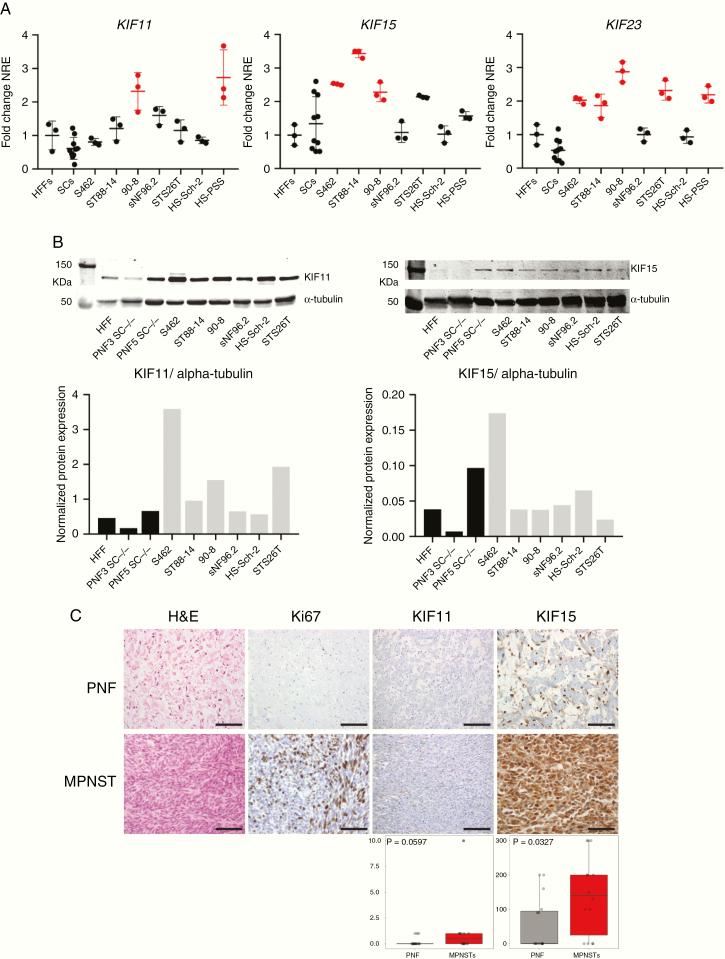
Mitotic kinesins KIF11, KIF15, and KIF23 are highly expressed in human MPNSTs and derived cell lines. A. RT-qPCR normalized relative expression (NRE) of *KIF11*, *KIF15*, and *KIF23* in CCD-1112Sk fibroblasts (HFFs), 10 human SC primary cultures, and 7 human MPNST cell lines. Expression values are plotted as the fold change NRE relative to the mean NRE in the HFF cultures. In red, those significantly overexpressed (one-way ANOVA). B. Top: Western blot of KIF11 and KIF15 proteins in HFFs, 2 PNF-SC cultures, and 6 MPNST cell lines. Bottom: Normalized KIF11 and KIF15 protein expression (vs. α-tubulin protein) in HFFs, 2 PNF-SC cultures, and 6 MPNST cell lines. C. Top: H&E staining and immunohistochemical expression of Ki67, KIF11, and KIF15 in a tissue microarray including 16 PNFs and 14 MPNSTs. Scale bars, 200 μm. Bottom: HScore values of KIF11 and KIF15 immunodetection in PNFs and MPNSTs (Mann–Whitney test).

After the collection of different types of genomic information obtained in MPNST and MPNST cell lines (Gel et al., manuscript in preparation) we observed an enrichment of mitotic kinesin members in altered chromosomal regions of MPNSTs. As these tumors contain hyperploid genomes and exhibit a high proliferation rate we reasoned that mitotic kinesins could play a functional role in MPNST survival. We considered KIF11, KIF15, and KIF23, which are involved at 2 different stages of mitosis (prophase and telophase). KIF11 is required for the separation of duplicated centrosomes during spindle formation in prophase^16^ and KIF15 for the maintenance of this spindle.^17^ KIF23 is involved in telophase and it is essential for cytokinesis.^18^We first assessed the expression of the 3 kinesins in our set of malignant (MPNSTs and MPNST cell lines) and benign (PNFs, SCs) samples. Their expression was assessed by Quantitative Reverse Transcription Polymerase Chain Reaction in a set of 7 MPNST cell lines, 10 benign SC primary cultures (8 DNF-SCs and 2 PNF-SCs), and the control HFF cell line CCD-1112Sk. Many of the MPNST cells, and not SCs, showed mRNA overexpression of these kinesin genes compared to control HFFs (Figure 1A). The expression of KIF11 and KIF15 protein was also studied by Western blot in 6 MPNST cell lines, 2 PNF-SCs, and HFFs. S462 cell line showed the highest expression of both kinesins among all cell lines. Overexpression of KIF11 was also found in most of the MPNST cells compared to benign and normal cells (Figure 1B). Furthermore, the immunodetection of KIF11 and KIF15 proteins in PNFs and MPNSTs was also assessed in a tissue microarray (Figure 1C). KIF15 expression was exclusively nuclear in most of the PNFs and mainly cytoplasmic in most MPNSTs. A significant overexpression of cytoplasmic KIF15 was found in MPNSTs compared to PNFs (Figure 1C). In our hands we were not able to reliably assess KIF23 protein expression in MPNST samples due to technical limitations.

### KIF15 and KIF23 Are Required for the Survival and Cell Cycle Progression of MPNST Cell Lines

**Figure 2.**
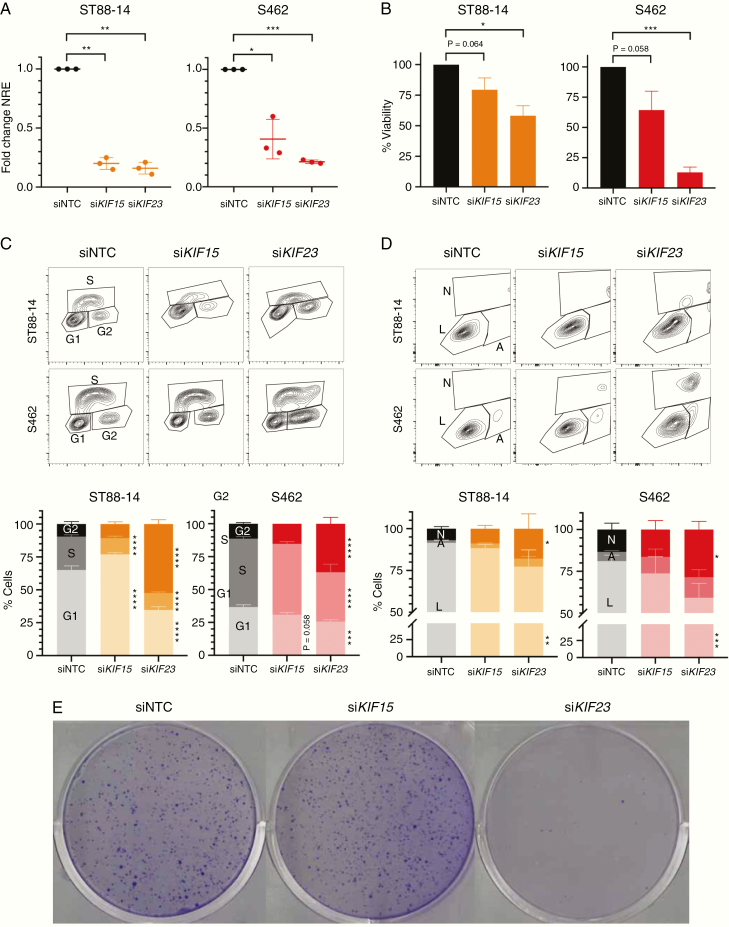
siRNA depletion of *KIF15* and *KIF23* affects viability and cell cycle progression of MPNST cells. A. RT-qPCR normalized relative expression of *KIF15* and *KIF23* 72h post-transfection of a non-targeting (siNTC) siRNA, si*KIF15*, and si*KIF23* pools in ST88-14 and S462 cell lines. Expression values are plotted as the fold change NRE for each condition relative to each NRE siNTC sample. B. Percentage of cell viability (relative to siNTC viability) after 72 h of transfection of siNTC, si*KIF15*, or si*KIF23* in ST88-14 and S462. C. Cytometry plots of EdU incorporation and PI staining (top) and percentage of cells in G1/G0, S, and G2/M cycles (bottom) after 72 h of transfection of siNTC, si*KIF15*, or si*KIF23* in ST88-14 and S462. D. Cytometry plots of Annexin V and bis-benzimide staining (top) and percentage of living (L), early apoptotic (A) and late apoptotic/necrotic cells (N; bottom) after 72 h of transfection of siNTC, si*KIF15*, or si*KIF23* in ST88-14 and S462. E. Colony formation assay in S462 cells after 15 days of transfection of siNTC, si*KIF15*, or si*KIF23*. All experiments were performed in triplicate. A *t*-test considering unequal variances was applied in A and B. A two-way ANOVA and a Tukey’s multiple comparisons test were applied in C and D (*P < .05, **P < .01, ***P < .001, ****P < .0001).

After expression analysis, an experimental framework was designed to suppress the function of the 3 kinesins KIF11, KIF15, and KIF23, considering expression and chemical suppression. We disrupted KIF15 and KIF23 using siRNA pool delivery in 2 MPNST cell lines: ST88-14 and S462. Expression knockdown was checked after 72 h of siRNA transfection by RT-qPCR and represented around 80% of expression reduction in all cases, except for *KIF15* in S462, which just a 60% of the reduction was achieved (Figure 2A). We then performed different in vitro functional assays (cell viability, cell proliferation, cell cycle and apoptosis analyses, and colony formation capacity) to evaluate the effect of *KIF15* and *KIF23* depletion in the tumorigenic properties of MPNST cells.

For KIF15 RNAi-mediated suppression, only a significant inhibition in cell proliferation was found in the ST88-14 cell line (Figure 2B–D). S462 cells transfected with siRNA pools targeting *KIF15* did not show a remarkable effect in the tumorigenic properties of this cell line, although its depletion in S462 cells was not as efficient as in ST88-14 cells. On the contrary, RNAi-mediated suppression of *KIF23* significantly inhibited both cell viability (Figure 2B) and proliferation (Figure 2C), promoted a G2/M cell cycle arrest (Figure 2C) and an apoptotic effect (Figure 2D) of MPNST cell lines. In addition, the *KIF23* knockdown also abrogated the colony formation capacity of S462 cells (Figure 2E).

### MPNST Cell Lines Are Sensitive to the KIF11 Inhibitors Ispinesib and ARRY-520

**Figure 3.**
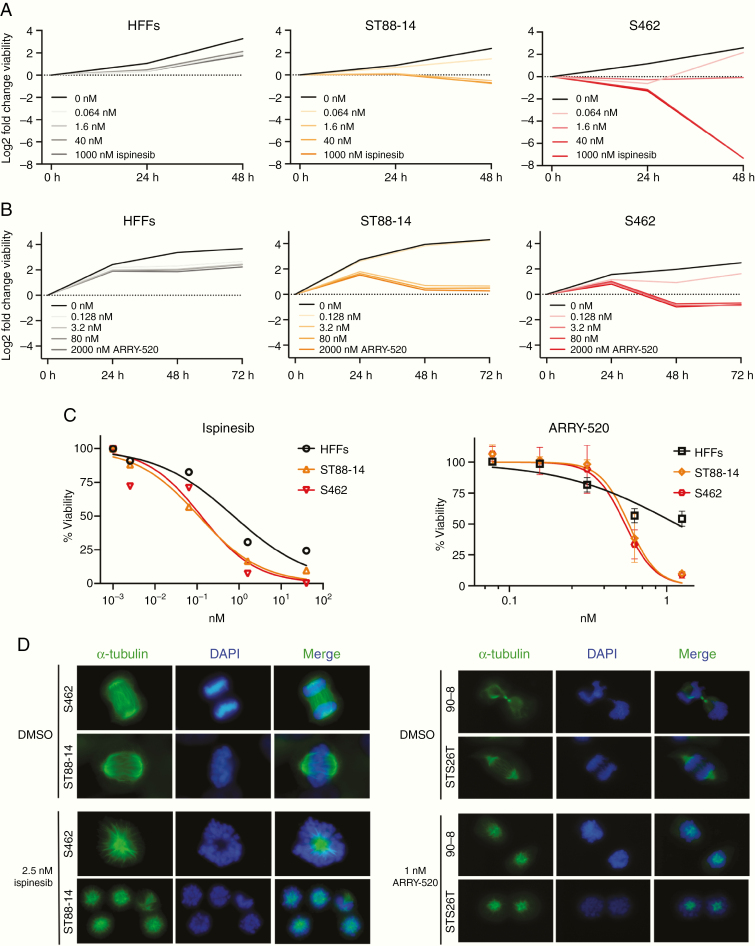
MPNST cell lines are sensitive to the KIF11 inhibitors ispinesib and ARRY-520. A. Log2 fold change viability of HFFs, ST88-14, and S462 at 24 h and 48 h versus 0 h of ispinesib treatment. B. Log2 fold change viability of HFFs, ST88-14, and S462 at 24 h, 48 h, and 72 h versus 0 h of ARRY-520 treatment. C. Viability curves after ispinesib (48 h) or ARRY-520 (72 h) treatment. The percentage of viable cells relative to dose 0 is plotted vs. KIF11 inhibitor dose. For ARRY-520 treatment mean ± SD viability is shown. IC_50_ values for each drug and cell line were calculated by applying a nonlinear regression model with the GraphPad Prism 8 software. D. Mitotic spindle immunostaining of MPNST cell lines after 24 h of ispinesib or ARRY-520 treatment. Normal functional bipolar spindles (top figures) were replaced by monopolar spindles (bottom figures). Representative images are shown (100×).

In contrast to KIF15 and KIF23, KIF11 can be targeted by several drug inhibitors.^19^ We suppressed the KIF11 function by using 2 of them, ispinesib and ARRY-520, in ST88-14 and S462 cells and assessed their viability. HFF benign fibroblasts were also used as a control nontumoral cell line to assess its sensitivity to KIF11 inhibition. In a first dose–response short time course, the viability of MPNST cells was strongly reduced at low ispinesib doses (1.6 nM), and as early as 24 h, and high doses were cytotoxic after 48 h for both MPNST cell lines, especially for S462 (Figure 3A). HFFs only showed mild cytostatic effects after 48 h of high doses (1000 nM). In the next dose–response experiment, cell number was determined after 48 h of ispinesib treatment and it was used to calculate the IC_50_ values (nM) for each cell line: ST88-14 (0.11), S462 (0.13), and HFFs (0.74). These values evidenced that MPNST cells were indeed more sensitive to ispinesib than nontumoral HFFs (Figure 3C, left). Preliminary experiments in a primary PNF-SC culture showed that high doses of ispinesib were mildly cytostatic for these cells (data not shown), as it has been described for 5 different KIF11 inhibitors (including ispinesib and ARRY-520) in 8 immortalized PNF-SC cultures.^20^

A new dose–response using ARRY-520 was also performed (Figure 3B). ST88-14 cells exhibited a stronger cytostatic effect at a lower dose (3.2 nM) just after 48 h and the effects on S462 at this time point and dose were cytotoxic (Figure 3B). HFFs showed a slight cytostatic effect after 72 h of a high dose of ARRY-520 (2000 nM; Figure 3B). Both MPNST cell lines showed a similar dose–response pattern with death in nearly all cells at the highest dose, which only killed half of the control HFF population (Figure 3C, right). Mean IC_50_ values (nM) for ARRY-520 in ST88-14, S462, and HFF were 0.57, 0.54, and 1.16, respectively (Figure 3C, right).

Both ispinesib and ARRY-520 inhibit the interaction between KIF11 and microtubules. This blockade prevents the formation of a functional bipolar mitotic spindle and the cell, consequently, dies. We checked the phenotypic effect of these drugs in the mitotic spindle formation of MPNST cells by performing immunofluorescence of α-tubulin after 24 h of treatment. In most MPNST dividing cells, inhibition of KIF11 with either ispinesib or ARRY-520 replaced the functional bipolar mitotic spindles by monopolar spindles, and the alignment of chromosomes at metaphase plate was abrogated (Figure 3D), as described elsewhere.^21^

### Co-targeting KIF11 and BRD4 With ARRY-520 and JQ1, Respectively, Synergistically Inhibits MPNST Cell Viability

**Figure 4.**
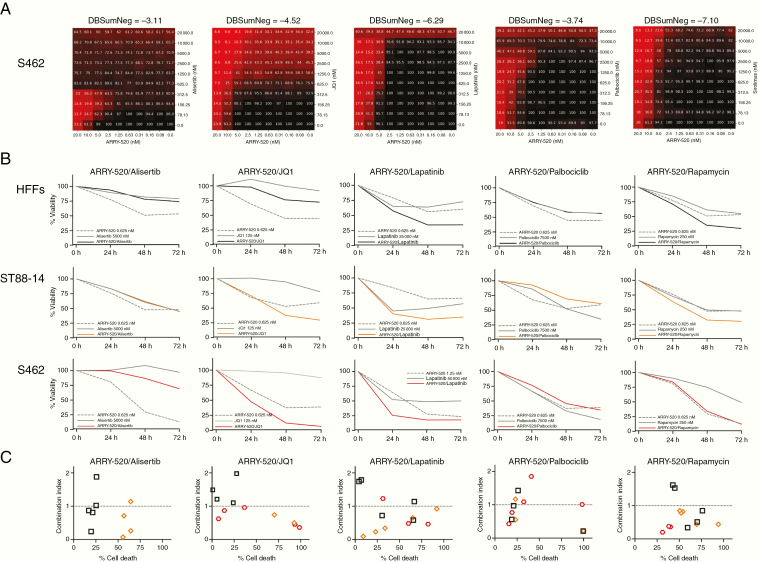
Co-targeting KIF11 (ARRY-520) and BRD4 (JQ1) synergistically kills MPNST cells. A. 10 × 10 matrix viability plots for the combination of ARRY-520 and each of the following drugs: alisertib (AURKAi), JQ1 (BRD4i), lapatinib (EGFR/ERBB2i), palbociclib (CDK4/6i), and rapamycin (aka, sirolimus; mTORi). B. Percentage of the viability of HFFs, ST88-14, and S462 cells at 0 h, 24 h, 48 h, and 72 h after combination treatments of ARRY-520 with alisertib, JQ1, lapatinib, palbociclib, or rapamycin. C. Combination Index (CI) plots of all 5 combined treatments at several doses showing the percentage of HFF, ST88-14, and S462 cell death versus the CI (CI > 1, antagonism; CI ≈1, additive effect; 0 < CI < 1, synergism).

To explore novel potential combined treatments for MPNSTs, we considered co-targeting KIF11 with other MPNST effectors in a mid-throughput in vitro analysis. We selected 20 inhibitors of described and proposed targets for MPNST therapy and designed combination treatments including an inhibitor of each of these targets with a KIF11 inhibitor (ispinesib or ARRY-520). We studied which of these combinations had a synergistic effect on killing the 2 MPNST cell lines S462 and sNF96.2 (Supplementary Figures S2–S5). According to viability values in the combination matrixes and the DBSumNeg values, several combinations involving KIF11 inhibitors were found to be synergistic (Figure 4A and Supplementary Figures S2–S5).

We selected 5 of these synergistic combined treatments to reproduce the results generated in the mid-throughput analysis, considering both their synergism (DBSumNeg) and the percentage of cell death (Figure 4A). We designed in vitro co-treatment using ARRY-520 in combination with alisertib, JQ1, lapatinib, palbociclib, or rapamycin, which respectively target AURKA, BRD4, EGFR/ERBB2, CDK4/6, and mTOR. Figure 4B shows the results of each dose–response for the 5 combined treatments in the 3 cell lines analyzed. In addition, we calculated the Combination Index (CI) value^22^ for the 5 dose–responses of each combined treatment to determine whether the combination had synergistic (0 < CI < 1), additive (CI ≈ 1), or antagonistic (CI > 1) effects (Figure 4C). We considered ARRY-520/JQ1 as the most efficient co-treatment among the combinations analyzed as it generated a synergistic effect (0 < CI < 1) at a high cell death percentage of ST88-14 and S462, and an antagonistic or additive effect (CI ≥ 1) at low cell death percentages of control HFFs (Figure 4B and C).

### Genetic Ablation of *KIF15* Enhanced MPNST Cells Sensitivity to KIF11 Inhibitors and to ARRY-520/JQ1 Combined Treatment

**Figure 5.**
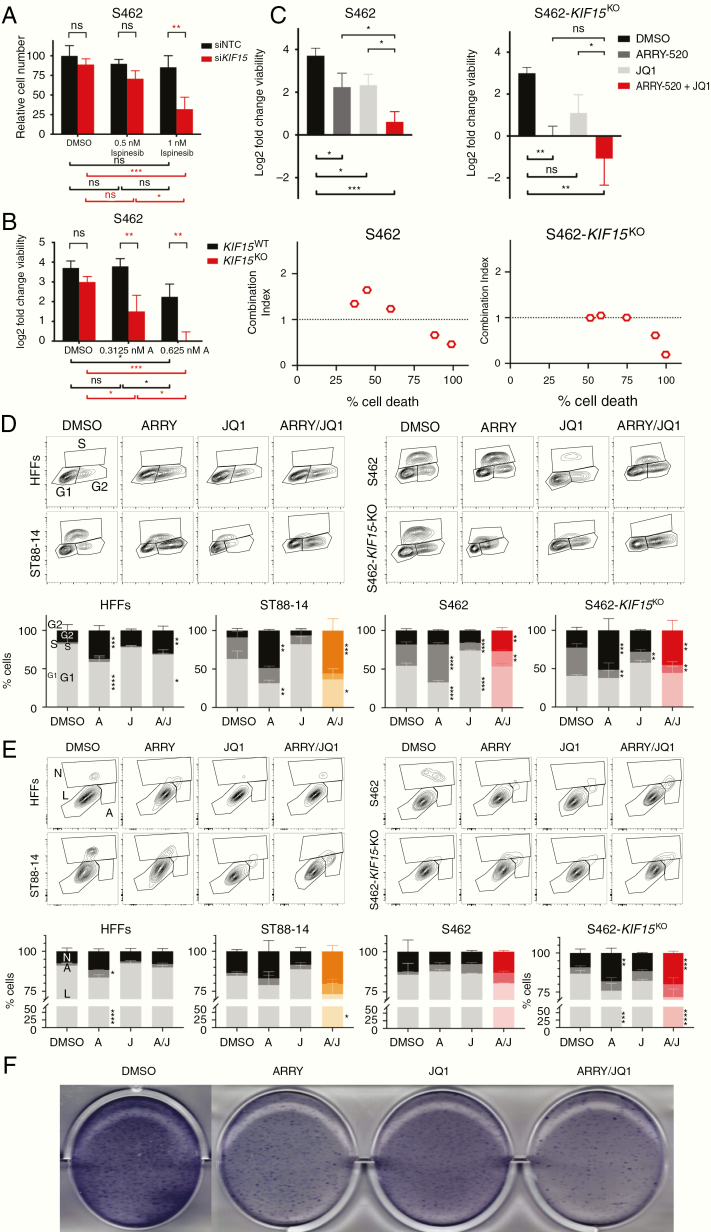
Combined suppression of KIF11 and KIF15 with BRD4 inhibition shows antitumoral in vitro effects in MPNST cells. A. Number of siNTC- and si*KIF15*-transfected S462 cells after 48 h of ispinesib treatment. Cell number is plotted relative to the number of cells in the siNTC sample at dose 0 nM (DMSO). B. Log2 fold change viability of *KIF15*^WT^ S462 and S462-*KIF15*^KO^ cells after 72 h of ARRY-520 treatment. C. Top: Log2 fold change viability of S462 and S462-*KIF15*^KO^ cell lines at 72 h versus 0 h of vehicle (DMSO), ARRY-520, JQ1, and combined ARRY-520/JQ1 treatments. Bottom: CI plots of S462 and S462-*KIF15*^KO^ showing the mean percentage of cell death versus the mean CI value for the ARRY-520/JQ1 treatment at 5 combined doses. D. Cytometry plots of EdU incorporation and PI staining (top) and cell cycle analysis (bottom) of HFFs, ST88-14, S462, and S462-*KIF15*^KO^ cells after 72 h of vehicle (DMSO), ARRY-520, JQ1, and combined ARRY-520/JQ1 treatments. E. Cytometry plots of Annexin V and bis-benzimide staining (top) and apoptosis analysis (bottom) of HFFs, ST88-14, S462, and S462-*KIF15*^KO^ cells after 72 h of vehicle (DMSO), ARRY-520, JQ1, and combined ARRY-520/JQ1 treatments. F. Colony formation assay of S462 cells after single agents and combined treatments. A one-way ANOVA was applied in C. A two-way ANOVA was applied in A, B, D, and E. A Tukey’s multiple comparisons test was applied in A–E. All experiments were repeated 3 times (*P < .05, **P < .01, ***P < .001, ****P < .0001).

It has been described that KIF11 and KIF15 show a partial functional redundancy: KIF15 can replace all essential functions of KIF11 in the creation of the bipolar spindle.^17^ We wanted to explore whether MPNST cells were more sensitive to KIF11 inhibition in a KIF15-deficient cellular background. In a first experiment, we assessed how a *KIF15* depletion affected the sensitivity to ispinesib by transfecting S462 cell line with either a siRNA pool targeting *KIF15* or an NTC siRNA (Figure 5A). After exposure to 1 nM ispinesib, si*KIF15*-transfected cells showed a significant reduction in viability compared to siNTC-transfected cells and also compared to vehicle (Figure 5A). In a second experiment, we studied the effect of a total loss of KIF15 in the sensitivity to the KIF11 inhibitor ARRY-520 by editing the *KIF15* gene with CRISPR/Cas technique. We generated a *KIF15*^KO^ S462 cell line and performed a dose–response treatment of ARRY-520 in this line and in *KIF15*^WT^ S462 control cells, also derived from a single clone (Figure 5B). *KIF15*^KO^ cells were significantly less viable than *KIF15*^WT^ cells when exposed to the KIF11 inhibitor (Figure 5B). In addition, ARRY-520/JQ1 co-treatment generated a significant reduction in the viability of both S462 and S462-*KIF15*^KO^ cells compared to vehicle (DMSO) (Figure 5C, top). The combination ARRY-520/JQ1 was clearly cytotoxic for KIF15-deficient S462 cells (Figure 5C, top). And in both S462 and S462-*KIF15*^KO^ cell lines, this combination was strongly synergistic (Figure 5C, bottom).

### Combined Inhibition of KIF11 and BRD4 Shows Synergistic Antitumoral Effects in MPNST Cell Lines, Especially in a KIF15-Deficient Background

We then used different in vitro functional assays to evaluate the effect of ARRY-520/JQ1 co-treatment in ST88-14, S462, and S462-*KIF15*^KO^ cells compared to control HFFs. In general, both single ARRY-520 and combined ARRY-520/JQ1 promoted a G2/M cell cycle arrest in all cell lines after 72 h. In ST88-14 and S462-*KIF15*^KO^ cells, the combined treatment produced a major G2/M arrest than in control HFFs (Figure 5D). ARRY-520/JQ1 co-treatment produced a reduction in proliferating EdU-positive S462 cells and a significant increase in G2/M cells, compared to vehicle. These 2 effects were increased in S462-*KIF15*^KO^ cells (Figure 5D). Regarding apoptosis, ARRY-520/JQ1 co-treatment produced an apoptotic-mediated cell death of all MPNST cells, especially ST88-14 and S462-*KIF15*^KO^. Interestingly, only the single ARRY-520 treatment, and not the combination, significantly increased the number of HFF apoptotic cells (Figure 5E). Finally, the colony formation capacity of S462 cells was reduced in single ARRY-520 and JQ1 treatments, but the colony number and extent were much lower when ARRY-520 and JQ1 were combined (Figure 5F).

## Discussion

This is the first study describing the expression of the mitotic kinesins KIF11, KIF15, and KIF23 in MPNSTs and their requirement for the survival of MPNST cell lines. We show that MPNST cell lines are sensitive to KIF11 inhibition and exhibit synergistic antitumoral effects when combined with BRD4 inhibition. In addition, we demonstrate that the loss of the genetic redundancy of KIF15 in MPNST cells increases their sensitivity to KIF11 inhibitors.

KIF11, KIF15, and KIF23 are overexpressed in several cancer types. In addition, the mitotic spindle kinesin KIF11 (also known as EG5) is required for tumor proliferation and has been proposed as a therapeutic target in glioblastoma,^23^ malignant mesothelioma,^24^ oral cancer,^25^ and breast cancer.^26^ Also the mitotic spindle kinesin KIF15 promotes the proliferation of pancreatic^27^ and bladder tumor cells.^28^ And the cytokinetic kinesin KIF23 (also known as MKLP1) promotes proliferation and has been suggested as a therapeutic target in glioma,^29^ malignant mesothelioma,^24^ and gastric cancer.^30^ MPNSTs bear hyperploid and highly rearranged genomes, a clear distinctive trait compared to PNFs or atypical neurofibromas.^3^ Typically, MPNSTs could exhibit tri- and tetraploid genomes. These are also aggressive tumors with a high mitotic index. In these contexts, the identification of an expression pattern of kinesins in MPNSTs and derived cells could represent a direct consequence of their gained genomic and hyperproliferative states. Thus, the suppression of mitotic kinesin functions may represent a vulnerability for MPNST cells. Our report demonstrates that KIF11, KIF15, and KIF23 are overexpressed in MPNST samples compared to their benign counterparts or nontumoral fibroblasts, as well as they are required for the survival and cell cycle progression of MPNST cell lines.

This study also shows that MPNST cell lines are more sensitive than benign control fibroblasts to the KIF11 inhibitors ispinesib and ARRY-520, which impair the formation of a functional bipolar mitotic spindle in MPNST cells in vitro. Both ispinesib and ARRY-520 have been tested in the clinics. Ispinesib (also known as SB-715992) reached phase II clinical trials in several cancers, although with null objective responses in nearly all cases.^31^ ARRY-520 (also known as filanesib), a more recent KIF11 inhibitor, has also been tested. In a phase II study, the single use of ARRY-520 in multiple myeloma generated a 16% response rate.^32^ Some of the reasons why KIF11 inhibitors did not achieve a successful clinical response as single agents are that nonresponsive tumors showed low KIF11 expression, had a low mitotic index, bore treatment-resistant mutations in KIF11, and exhibited KIF15 activity.^31^

There is a partial functional redundancy between the 2 studied spindle proteins: KIF15 can replace all essential functions of KIF11 in the creation of the bipolar spindle^17^ and a KIF15-dependent resistance to KIF11 inhibition has already been described in vitro.^33,34^ In our study, either a partial or a total genetic suppression of *KIF15* conferred the MPNST cell line S462 to increase its sensitivity to KIF11 inhibition, supporting a functional role for both KIF11 and KIF15 in the survival of MPNST cells. This result also supports the rationale that targeting proteins that have a genetic redundancy or a functional compensation^35^ may represent a promising therapeutic strategy for MPNSTs. A recent study showed that combined inhibition of KIF11, either with ispinesib or ARRY-520, and KIF15, with the novel inhibitor KIF15-IN-1, synergistically and efficiently killed HeLa cells, constituting a potential strategy for overcoming chemotherapeutic resistance.^36^ The constant development and use of novel kinesin inhibitors in preclinical and clinical studies in other cancers reinforces the potential use of kinesin inhibitors for treating MPNSTs. The increased impairment of MPNST cell viability after the suppression of both KIF11 and KIF15 mitotic spindle proteins suggests that targeting their redundant function may represent a therapeutic vulnerability for these tumors.

Finally, it has been described that loss of PRC2 confers sensitivity to BRD4 inhibition in MPNST.^7^ Several combined therapies considering the BRD4 inhibitor JQ1 and other MPNST targets, such as MEK,^7^ and mTOR^37^ have been proposed. Our report demonstrates that MPNST cells exposed to a combined ARRY-520/JQ1 treatment are much less viable than fibroblasts, and co-targeting KIF11 and BRD4, for a given combined dose, synergistically kills almost all MPNST cells, especially those KIF15-deficient and, interestingly, only kills about half of the control fibroblasts, and in an antagonistic manner.

In conclusion, our study shows that mitotic kinesins are potential therapeutic vulnerabilities for MPNSTs and demonstrates that the combined suppression of KIF11 and KIF15 together with BRD4 inhibition exhibit in vitro synergistic antitumoral effects in MPNST cells. Our in vitro results evidence that, although further in vivo experiments are still mandatory, a simultaneous suppression of KIF11, KIF15, and BRD4 may be a potential therapy for MPNSTs.

## Funding

This work has been supported by the Instituto de Salud Carlos III [PI14/00577, PI17/00542], Plan Estatal de I+D+I 2013-2016 and co-financed by FEDER program; and the Generalitat de Catalunya [2014-SGR-338, 2017-SGR-496], CERCA Program.

## Supplementary Material

vdz061_suppl_Supplementary_Figure_Legends

vdz061_suppl_Supplementary_Figure_S1

vdz061_suppl_Supplementary_Figure_S2

vdz061_suppl_Supplementary_Figure_S3

vdz061_suppl_Supplementary_Figure_S4

vdz061_suppl_Supplementary_Figure_S5

vdz061_suppl_Supplementary_Methods
