# Supplementary material for: KIF11 and KIF15 mitotic kinesins are potential therapeutic vulnerabilities for malignant peripheral nerve sheath tumors
Source: Neurooncol Adv. 2020 Jan 4;2(Suppl 1):i62–74. doi: 10.1093/noajnl/vdz061 (PMC7317059; doi:10.1093/noajnl/vdz061)
Supplement: vdz061_suppl_Supplementary_Figure_Legends [file vdz061_suppl_supplementary_figure_legends.docx]

***Supplementary Figure Legends***

**Figure S1.** Western blot of KIF11, KIF15 and α-tubulin in S462 cell line, the S462 single cell derived clone (S462*) and several CRISPR/Cas *KIF15*-edited S462* clones. S462* KIF15#2D cells did not express KIF15 and were used for further experiments.

**Figures S2-S5**. 10x10 matrix viability plots for the combination of ispinesib (S2, S3) or ARRY-520 (S4, S5) with each of the following 20 drugs (and inhibited targets): alisertib (AURKAi), barasertib (AURKBi), sepantronium bromide (BIRC5i), JQ1 (BRD4i), palbociclib and ribociclib (CDK4/6i), plerixafor hydrochloride (CXCR4i), lapatinib (EGFR/ERBB2), vorinostat (aka SAHA, HDAC1/3i), tanespimycin (HSP90i), imatinib mesylate (KIT/PDGFR/ABLi), PD-03259010 (MEKi), rapamycin (aka sirolimus; mTORC1i), AZD8055 (mTORC1/2i), doxorubicin (TOP2Ai), cabozantinib (VEGFR2/MET/MNKi), chloroquine (authophagy inhibitor), XAV-939 (TKNKSi), Wnt-C59 (PORCNi) and vismodegib (SMOi). Two MPNST cell lines were used: S462 (S2, S4) and sNF96.2 (S3, S5). The DBSumNeg values, indicatives of the synergistic effect are also included on top of every combination matrix.
