# Supplementary figures and images for: KIF11 and KIF15 mitotic kinesins are potential therapeutic vulnerabilities for malignant peripheral nerve sheath tumors
Source: Neurooncol Adv. 2020 Jan 4;2(Suppl 1):i62–74. doi: 10.1093/noajnl/vdz061 (PMC7317059; doi:10.1093/noajnl/vdz061)

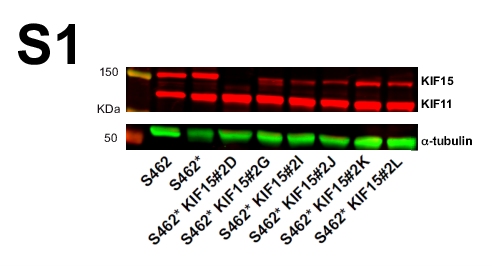

Supplement: vdz061_suppl_Supplementary_Figure_S1 [file vdz061_suppl_supplementary_figure_s1.png]

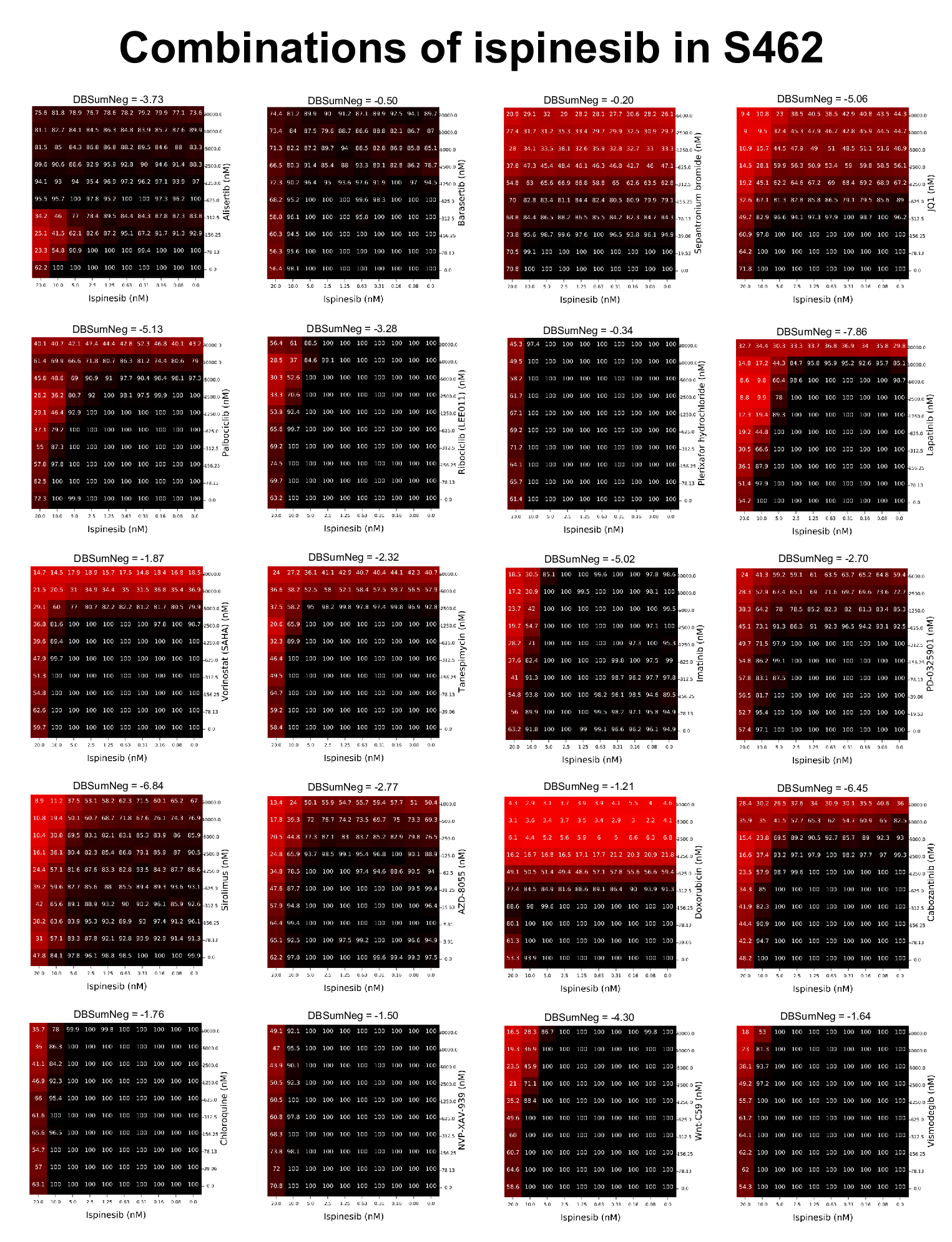

Supplement: vdz061_suppl_Supplementary_Figure_S2 [file vdz061_suppl_supplementary_figure_s2.png]

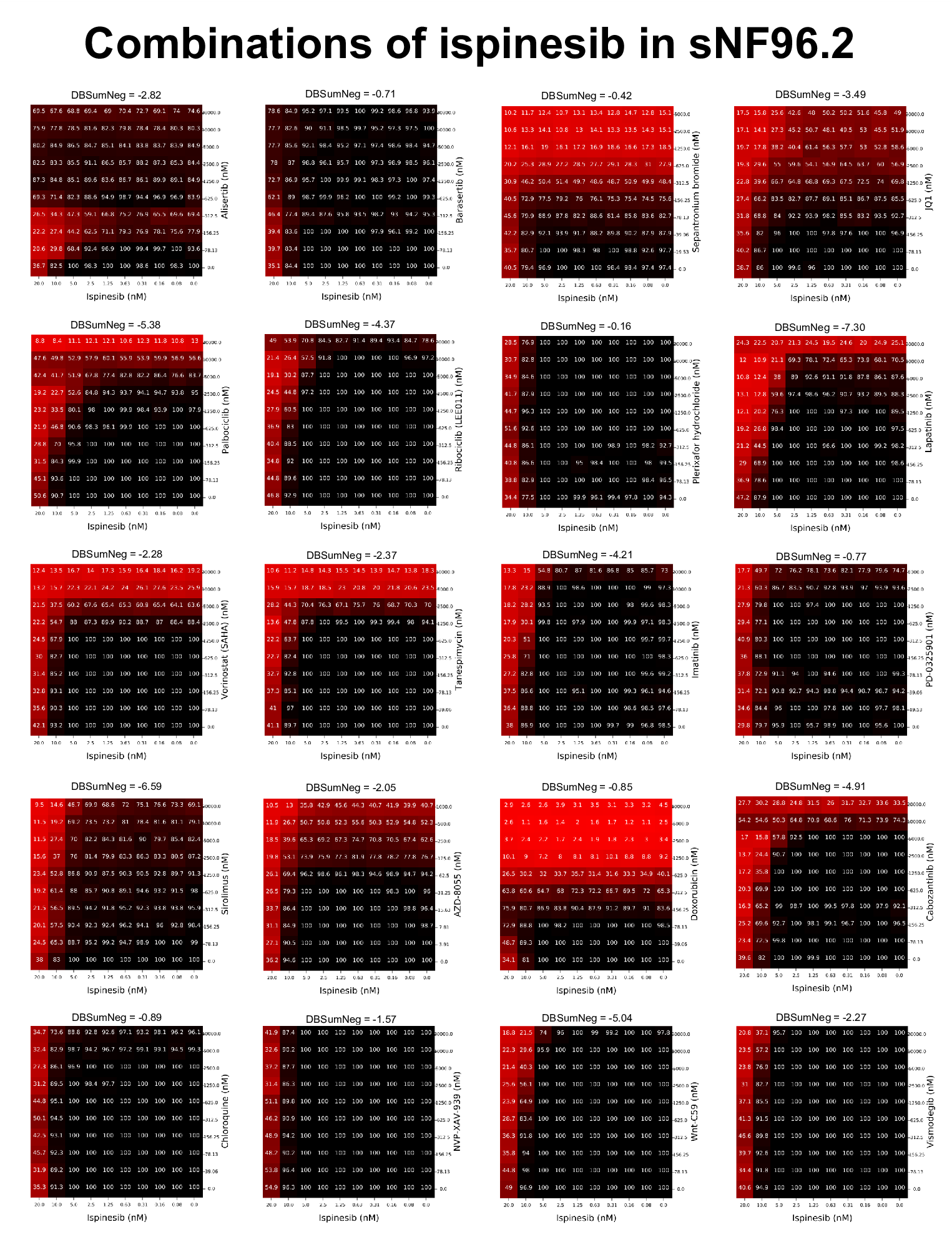

Supplement: vdz061_suppl_Supplementary_Figure_S3 [file vdz061_suppl_supplementary_figure_s3.png]

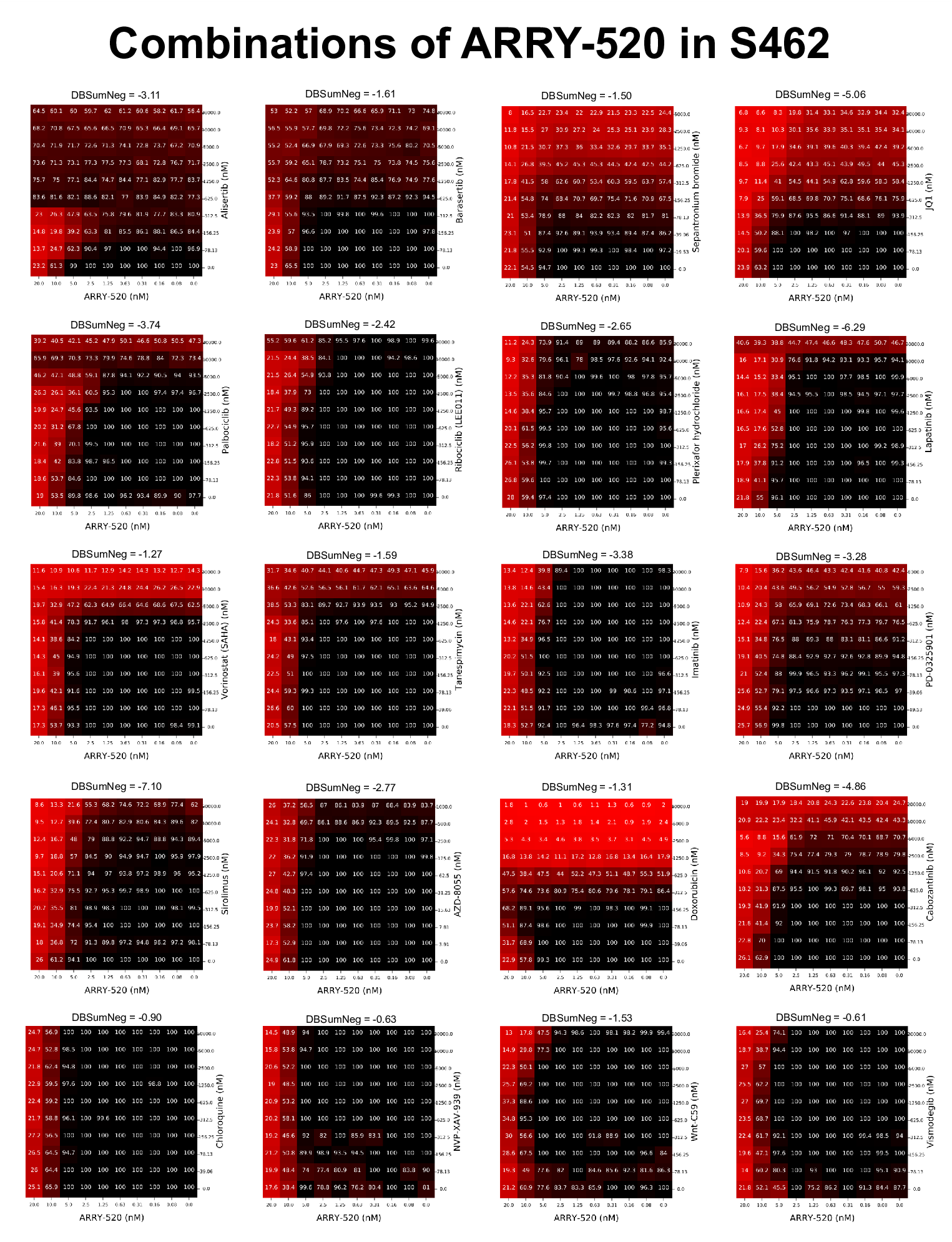

Supplement: vdz061_suppl_Supplementary_Figure_S4 [file vdz061_suppl_supplementary_figure_s4.png]

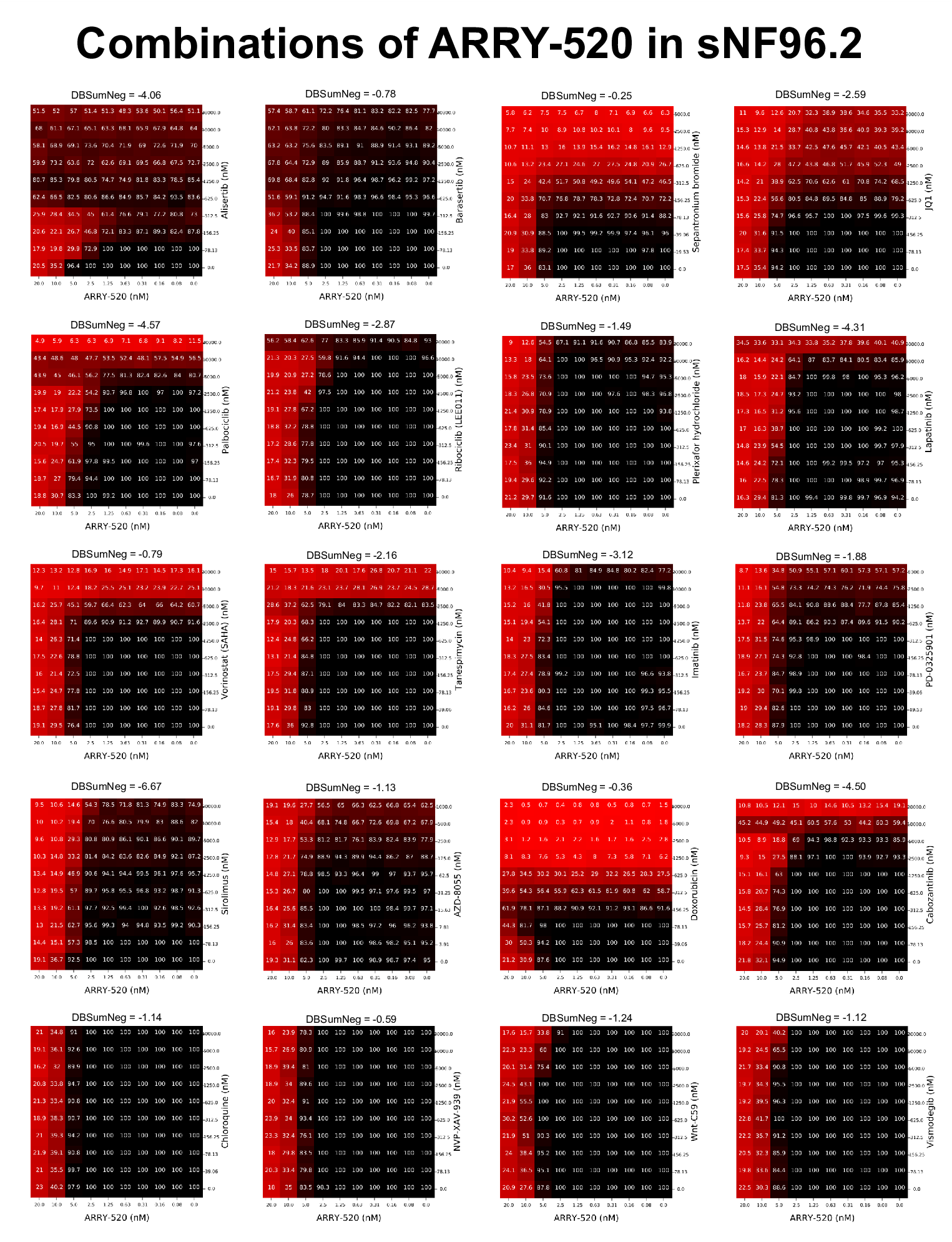

Supplement: vdz061_suppl_Supplementary_Figure_S5 [file vdz061_suppl_supplementary_figure_s5.png]
