# Supplementary material for: KIF11 and KIF15 mitotic kinesins are potential therapeutic vulnerabilities for malignant peripheral nerve sheath tumors
Source: Neurooncol Adv. 2020 Jan 4;2(Suppl 1):i62–74. doi: 10.1093/noajnl/vdz061 (PMC7317059; doi:10.1093/noajnl/vdz061)
Supplement: vdz061_suppl_Supplementary_Methods [file vdz061_suppl_supplementary_methods.docx]

***Supplementary Extended Methods***

**Cell lines and primary cultures**

The MPNST cell lines S462, ST88-14, sNF96.2, 90-8, STS-26T, HS-Sch-2 and the human foreskin fibroblast (HFF) cell line CCD-1112Sk (ATCC®) were cultured under standard conditions (37ºC, 5% CO2) with High Glucose DMEM with sodium pyruvate (Biowest) supplemented with 10% FBS (Biowest) and 2mM L-Glutamine (Life technologies), and passaged using trypsin-EDTA (Life technologies).

SC primary cultures were isolated from DNFs and PNFs and cultured as previously described^1^. Briefly, surrounding fat and skin tissue were removed from tumors, which were later cut into 1-2 mm pieces and cryopreserved (90% FBS + 10% DMSO) until used. Then for SC isolation, tumor pieces were thawed and digested with 160 U/mL collagenase type 1 and 0.8 U/mL dispase (Worthington) for 16h at 37^o^C. Dissociated cells were then seeded on dishes previously coated with 0.1 mg/mL poly-L-lysine (Sigma) and 4 mg/mL laminin (Gibco), and maintained in Schwann Cell Medium (SCM) at 37ºC, 10% CO2. SCM is composed of High Glucose DMEM with sodium pyruvate (Biowest), 10% FBS (Biowest), penicillin/streptomycin (Gibco), 0.5 mM 3-iso-butyl-1-methilxantine (Sigma), 2.5 mg/mL insulin (Sigma), 10 nM heregulin-β1 (PeproTech), and 0.5 mM forskolin (Sigma). One day after plating, culture medium was replaced by SCM without forskolin for an additional 2–3 days. This procedure was repeated in cycles and cells were passaged when needed with trypsin-EDTA (Life technologies). SC purity was assessed by performing S100β staining as described previously^1^.

**Immunohistochemistry**

A tissue microarray (TMA) was used to check the expression of KIF11 and KIF15. Clinical and pathological characteristics of 16 PNFs and 14 MPNSTs from patients diagnosed at the Vall d’Hebron Hospital between 2002 and 2015 were included in the study. TMAs were performed including three 2mm samples from each lesion using the TMA Master from 3DHistech.

FFPE tissue 3 μm thick sections were incubated with 1:50 anti-KIF11 antibody (Proteintech), 1:50 anti-KIF15 antibody (Proteintech) or 1:50 Ki67 (Ventana Medical Systems). Immunohistochemical staining was performed on the Ventana Benchmark XT Automated IHC Stainer using the Ventana ultraView Universal DAB Detection Kit (Ventana Medical Systems Inc). In brief, after deparaffinization with Ventana EZ Prep solution, antigen retrieval was performed using Ventana Tris-based buffer solution CC1 at standard temperature for 60 minutes. Endogenous peroxidase was blocked with ultra-View inhibitor, 3% hydrogen peroxide, for 4 minutes at 37^o^C. After rinsing, slides were incubated at 37^o^C for 30 min with KIF11 antibody, 1 h with KIF15 antibody and 16 min with Ki-67 antibody. Primary antibodies-horseradish peroxidase-labeled antibody complex were visualized using diaminobenzidine tetrahydrochloride chromogen. Slides were then counterstained for 8 min with hematoxylin and for 4 min with bluing reagent. Appropriate positive and negative controls were included within the study sections.

Evaluation of immunohistochemical staining were evaluated as follows: intensity (0-3) and percentage of positive cells (0-100) were evaluated for KIF11 and KIF15. HScore (0-300; intensity per percentage of positive cells) was calculated in both cases.

**RT-qPCR**

RNA was extracted from cells using Tripure Isolation Reagent (Roche) according to the manufacturer’s instructions. A NanoDrop® ND-1000 spectrophotometer (Thermo Scientific) was used to quantify RNA and to measure purity and quality. For the retrotranscription (RT) reaction, 1 μg of RNA was mixed with 1 μL of random hexamers (Life technologies), 1 μL of dNTPs mix (10mM each; vWR), 4 μL of 5X First-Strand buffer (Life technologies), 2 μL of 0.1M DTT (Life technologies) and 0.8 μL of Superscript III reverse transcriptase enzyme (Life technologies). Conditions for the RT reaction in the thermocycler (2720 Thermal Cycler, Applied Biosystems) were as follows: 25°C for 12 min, 42°C for 50 min and 70ºC for 15 min. Finally, cDNA sample was 1:10 diluted with RNAse-free water and used as a template for the qPCR experiments.

Primers and probes for the RT-qPCR assay were designed with Roche Universal ProbeLibrary (UPL) technology. When possible, an intron spanning design was chosen, which considers both primers from the pair in different but contiguous exons, thus avoiding amplification of potential genomic DNA present in the sample. The design of each of the primers (desalted and purified; Life technologies) was subjected to an *in silico* PCR and BLAT search analysis to evaluate their specificity, prior qPCR experiments.

qPCR experiments were performed in a Light-Cycler® 480 Real-Time PCR System with white Multiwell Plate 384 plates (Roche Diagnostics). Conditions for amplification were as follows: 95°C for 10 min; 45 cycles of 95°C for 10 s, 60°C for 30 s, and 72°C for 1 s; and 40°C for 30 s. The linear dynamic range (LDR) and efficiency (E) of the primers were evaluated. Each reaction in all experiments included 2 μL of diluted cDNA template, 4 μL of 2X LightCycler 480 Probes Master Mix (Roche Diagnostics), 0.1 μmol/L UPL probe, and 0.5 or 0.75 μmol/L of each primer (depending on the gene), in a total volume of 8 μL. PCR reactions for each primer set and sample were performed in triplicate and each set of PCR assays included both negative controls without template. In addition, a calibrator sample was also included in triplicate in every assay.

A Microsoft Excel spreadsheet was used to analyze qPCR data for relative expression calculations as described^2^. We used formulas from the qBase relative quantification framework^3^, which are based on the Pfaffl method^4^. In brief, we averaged the 3 quantification cycle (Cq) numbers obtained from each triplicate with the second-derivative maximum method in the Light-Cycler® 480 software. We then calculated the ΔCq value for the difference between the unknown sample and the calibrator sample (ΔCq = Cq_unknown_ – Cq_calibrator_). The relative quantity (RQ) was later calculated as: RQ = E^–ΔCq^. We calculated the normalized relative expression (NRE) as: NRE = RQ/NF, where RQ is the relative quantity and NF is the normalization factor. The NF is, for a particular sample, the geometric mean of the RQ values for the 2 selected reference genes for expression normalization. We selected *HMBS* and *TBP* genes as reference genes, as they have been described to be suitable reference genes for NF1-associated samples, such as SCs^5^.

The sequences of the primers used for the RT-qPCR experiments are:

*KIF11*-F: 5’- gagcagattgtagaattgattgaaaa *KIF11*-R: 5’- ggtcagatttacactggtcaagttc

*KIF15*-F: 5’- tcattctacccaaatgcagga; *KIF15*-R: 5’- ttcaaggacattcaactgtgaga

*KIF23*-F: 5’- gaaccaaatgtatggaactaacaaga; *KIF23*-R: 5’- cacacgatcatccgcactt

*HMBS*-F: 5’- tcctgaggcacctggaag; *HMBS*-R: 5’- ttgtatgctatctgagccgtcta

*TBP*-F: 5’- aggaattgaggaagttgctgag; *TBP*-R: 5’- cgctggaactcgtctcacta.

The UPL probes (Roche) used were:

#17 (*KIF11*), #4 (*KIF15*), #60 (*KIF23*)

#25 (*HMBS*), #67 (*TBP*)

**Protein extraction and Western blot**

For protein extraction, cells were washed with cold PBS twice and lysed with RIPA buffer (50 mM Tris-HCl pH 7.4, 150 mM NaCl, 1mM EDTA, 0.5% IGEPAL® CA-630) supplemented with 3mM DTT (Roche), 1 mM PMSF (Fluka), 1 mM sodium orthovanadate (Sigma), 5 mM NaF (Honeywell), 10 μg/ml leupeptin (Sigma), and 5 μg/ml aprotinin (Sigma). Cell lysates were then boiled with Laemmli buffer and 20-30 µg of protein were subjected to SDS-PAGE and transferred on PVDF membranes (18 h, 90 mA at 4ºC). Membranes were blocked with Odyssey Blocking Buffer-PBS (LI-COR) and incubated with primary antibodies 1:500 anti-KIF11 (Proteintech) or 1:500 anti-KIF15 (Proteintech) at 4ºC overnight, washed, incubated with 1:5,000 anti-α-tubulin (Sigma-Aldrich) for 1 h at room temperature and washed again. Membranes were finally incubated with 1:15,000 IRDye 680LT and 1:25,000 IRDye 800CW secondary antibodies (LI-COR) for 1 h at room temperature, washed and scanned using the Odyssey Imaging System (LI-COR).

**siRNA transfection**

For the siRNA experiments we selected Dharmacon siGENOME SMARTpool technology because they are composed of a mixture of 4 different siRNAs targeting a particular gene of interest in a single reagent. Except for the cell viability assay in ST-8814 cell line, 20,000 (S462) or 60,000 (ST-8814) cells were plated for each condition in a well of a 12-well plate and incubated overnight with 400 μL of supplemented DMEM. A mixture of 0.5 μL of 20 μmol/L siRNA (20 nM final concentration) and 1 μL of lipofectamine diluted in 100 μL of opti-MEM (Life technologies) was added to each well. After 24 h of transfection, medium was replaced with 1 mL of supplemented DMEM. Only for the anchorage-independent growth assay, cells were harvested 24 h post-transfection and used for the assay (see below). For the treatment of siRNA-transfected cells, they were treated 48 h post-transfection. For RNA extraction, and the cell proliferation, apoptosis and cell counting *in vitro* functional assays, cells were let to grow for extra 48 h and were harvested, 72 h post-transfection, with trypsin-EDTA (Life technologies). For the cell viability assay in ST-8814 cell line, 1,500 cells per well were plated in a 96-well plate and incubated overnight with 100 μL of supplemented DMEM. A mixture of siRNA pool (20 nmol/L) and lipofectamine (0.25 μL) diluted in 25 μL of opti-MEM (Life technologies) was added in tetraplicate and incubated for 24 h in standard conditions. After that, medium was replaced with 100 μL of supplemented DMEM and cells were let to grow for extra 48 hours. Moreover, 100 μL of supplemented DMEM were also added in tetraplicate in empty wells with no cells, as the blank condition.

**CRISPR/Cas edition**

For generating the CRISPR constructs, we selected the pX330-U6-Chimeric_BB-CBh-HSpCas0 vector (Addgene)^6^ and the pRGS2 surrogate reporter vector (Labomics)^7^. Briefly, we ligated the gRNA sequence (5’- gattacagcacttgtcgacg**)** to the digested plasmids, which were transformed to bacterial competent cells, and a Miniprep pDNA extraction (Macherey-Nagel) was performed to obtain the vectors containing the gRNAs of interest.

S462 cells derived from a single cell clone were seeded in 6-well plates and transfected with both pX330 and PRGS2 vectors with Lipofectamine 2000 (Life Technologies). Three days after transfection, cells were sorted using the FACSAria II (BD Biosciences) and single RGF+GFP+ cells were plated in 96-well plates, and cultures in standard conditions. Total protein was extracted from some viable edited clones and *KIF15* expression was checked by Western blot (**Supplementary Figure S1**). One of the viable S462 clones was found to be KIF15-deficient and was used together with *KIF15*^WT^ S462 clonal cells for single and combined treatments (**Supplementary Figure S1**).

***In vitro* single drug treatment**

For the dose-response time-course experiment, ispinesib (Selleckchem) treatment was performed in 96-well plates that were previously seeded with 1,000 cells (S462) or 1,500 cells (ST88-14 and HFFs) per well and incubated overnight with 100 μL of supplemented DMEM. At 0 h timepoint, media was replaced with 100 μL of supplemented DMEM containing vehicle (DMSO), 0.064, 1.6, 40 or 1000 nM ispinesib (each concentration in tetraplicate). Cells were allowed to grow for 48 h and cell viability was measured with XTT assay (see below) at 0 h, 24 h and 48 h timepoints. For the calculation of the half maximal inhibitory concentration (IC50), S462 (100,000 per well), ST88-14 and HFFs (150,000 per well each) were plated in a 6-well plate and incubated with 1 mL of supplemented DMEM. The following day media was replaced with 1 mL of supplemented DMEM containing vehicle (DMSO), 0.003, 0.064, 1.6 or 40 nM ispinesib (each concentration in triplicate). Cells were let to grow for 48 h, trypsinized, resuspended in PBS, and counted with the Countess^TM^ Automated Cell Counter (Life technologies). For the dose-response of ispinesib treatment after *KIF15* depletion, a combination of siRNA transfection for the expression knockdown of *KIF15* (Dharmacon) and the NTC siRNA (Dharmacon), and a chemical inhibition of KIF11 protein with ispinesib (Selleckchem) was performed in S462 cell line. In this case, 250,000 S462 cells were plated in a 10 cm plate and incubated overnight with 5 mL of supplemented DMEM. A mixture of 6.25 μL of 20 μmol/L siRNA pool (targeting *KIF15* or a NTC) and 12.5 μL of lipofectamine RNAiMAX (Life technologies) diluted in 1.25 mL of opti-MEM (Life technologies), 20 nM siRNA final concentration, was added to each plate. After 24 h of transfection, cells were harvested and counted. Then, 100,000 cells per well were plated on a 6-well plate and incubated with 2 mL of supplemented DMEM. The following day media was replaced with 2 mL of supplemented DMEM containing varying concentrations of ispinesib. Cells were let to grow for 48 h, trypsinized, resuspended in PBS and counted with the Countess^TM^ Automated Cell Counter (Life technologies). Viability was calculated for each dose as the number of cells of this particular dose with respect to vehicle (DMSO) after 48 h of treatment.

Cell viability was measured during ARRY-520 treatment using the RealTime-Glo™ MT Cell Viability Assay (Promega). ARRY-520 treatment was performed in 96-well plates previously seeded with 1,000 cells (S462 and S462-*KIF15*^KO^) or 1,500 cells (ST88-14 and HFFs) per well and incubated overnight with 100 μL of supplemented DMEM. For the dose-response time-course of HFF, ST88-14 and S462 cells, media was replaced at 0 h timepoint with 100 μL of supplemented DMEM containing vehicle (DMSO), 0.128, 3.2, 80 or 2000 nM (each concentration in triplicate). For the IC50 calculation of S462, ST88-14 and S462 and for the dose-response time-course of S462 and S462-*KIF15*^KO^ cells, media was replaced at 0 h timepoint with 100 μL of supplemented DMEM containing vehicle (DMSO), 0.078, 0.156, 0.313, 0.625 or 1.25 nM (each concentration in triplicate). In all experiments, cells were allowed to grow for 72 h and cell viability was measured at 0 h, 24 h, 48 h and 72 h.

***In vitro* combined drug treatment**

We performed a dose-response experiment where HFFs, ST88-14 and S462 cells were treated with vehicle (DMSO), six concentrations of single ARRY-520, six concentrations of the single second drug, and six combined concentrations of ARRY-520 with the second drug, in a time course. The drugs used for the combinations with ARRY-520 were alisertib (AURKAi), JQ1 (BRD4i), lapatinib (EGFRi), palbociclib (CDK4/6i) and rapamycin (mTORi). Cell viability was measured at 0h, 24h, 48h and 72h of treatment using the RealTime-Glo™ MT assay.

We also studied the effect of a 0.625 nM ARRY-520 and 12.5 μM JQ1 co-treatment in S462 and S462-*KIF15*^KO^ viability (RealTime-Glo™ MT assay). And the effect of a 1.25 nM ARRY-520 and 500 nM JQ1 co-treatment in cell proliferation and cell cycle by cytometry analysis of EdU incorporation, and in cell apoptosis by cytometry analysis of Annexin V staining of HFFs, ST88-14, S462 and S462-*KIF15*^KO^. All cells were exposed to vehicle (DMSO), 1.25 nM single ARRY-520, 500 nM single JQ1 or 1.25 nM/500 nM combined ARRY-520/JQ1, and cytometric analyses were performed after 72h of treatment.

**Cell viability analysis**

Viability of siRNA-transfected ST88-14 cells, and viability of HFFs, ST88-14 and S462 during ispinesib treatment was analyzed using the XTT assay (Roche). XTT is a tetrazolium salt that, in the presence of an electron-coupling reagent, produces a water-soluble formazan salt. Tetrazolium salts are cleaved to formazan by the succinate-tetrazolium reductase system in the respiratory chain of the mitochondria. Therefore, the amount of formazan dye generated directly correlates to the number of metabolically active cells in the culture. Formazan dye can be quantified using a multi-well spectrophotometer reader. After 72h of transfection, 50 μL of XTT labeling mixture (XTT labeling reagent and electron-coupling reagent 50:1) were added to each well, and the plates were incubated in a cell culture incubator for 1-2 hours in standard conditions. After that, absorbances at 492 nm (A492) and 690 nm (A690) were measured for every well with a 96-well plate spectrophotometer reader (Spectra Max 340PC). Then, the A492 – A690 value was calculated for every single well, including the blank measurement. Viability was then calculated, in arbitrary units, for a particular well as (A492 – A690)_well_ – (A492 – A690)_blank_.

Viability of siRNA-transfected S462 cell line was measured by automated cell counting. Cells from each condition were harvested 72h post-transfection and diluted with PBS. A mixture of 10 μL of cell suspension and 10 μL of trypan blue dye (Nanoentek) was added to an EVE cell counting slide (Nanoentek) in duplicate. The slide was introduced into the Countess^TM^ Automated Cell Counter (Life technologies) and the number of cells was then calculated.

Viability of HFFs, ST88-14, S462 and during single ARRY-520 and combined treatments was analyzed with the RealTime-Glo™ MT assay (Promega). This assay is based on a non-lytic NanoLuc Luciferase reaction that occurs in the culture medium. The MT Cell Viability Substrate diffuses into cells where it is reduced to form a NanoLuc Substrate, which exits the cell and is used rapidly by NanoLuc Luciferase in the media. Only metabolically active cells are able to reduce the NanoLuc substrate, and light production is proportional to the number of live cells in culture. Dead cells cannot reduce the substrate. NanoLuc Luciferase and MT Cell Viability Substrate were added to High Glucose DMEM culture medium together with the drugs or vehicle in 96-well plates, according to manufacturer’s instructions. Light intensity was measured with a luminometer plate reader at 0h, 24h, 48h and 72h.

**Cell proliferation and cell cycle analysis**

Proliferating cells and cell cycle phases were analyzed using the Click-iT® EdU Alexa Fluor® 488 Flow Cytometry Assay Kit (Life Technologies) in combination with propidium iodide (PI, Sigma) staining. EdU (5-ethynyl-2’-deoxyuridine) is a nucleoside analogue to thymidine that is incorporated into DNA during active DNA synthesis. Detection with this technology is based on a click reaction: a copper catalyzed covalent reaction between an azide and an alkyne. In this assay, the alkyne is found in the ethynyl moiety of EdU, while the azide is coupled to an Alexa Fluor® 488 dye.

As a summarized protocol, cells were incubated with 20 μmol/L EdU for 1 h prior trypsinization. Then, harvested cells from each condition were washed with 1% BSA-PBS, fixed with 4% p-formaldehyde, permeabilized with a saponin-based buffer, incubated for 30 min with the Click-iT EdU reaction cocktail (containing both Alexa Fluor® 488-azide and CuSO_4_ needed for the click reaction), and then washed again. A final incubation with 15 mg/mL PI in citrate buffer for 30 min was performed. Finally, 10,000 cells per condition were analyzed with the BD LSRFortessa SORP cytometer using the B530-A and B695-A lasers for the measurement of the Alexa Fluor® 488 dye (EdU positive cells) and PI (DNA content), respectively, in the Cytometry Service from the Germans Trias i Pujol Research Institute.

**Apoptosis analysis**

Apoptotic and necrotic cells were analyzed using the Annexin-V-Alexa Fluor® 568 antibody (Roche and Life Technologies) and bis-benzimide dye (also known as Hoechst 33342, Sigma) staining. Annexin-V is a cytoplasmic protein with a high affinity for phosphatidylserine. In the early stages of apoptosis, there is a flip-flop translocation of phosphatidylserine from the inner layer of the cell membrane to the outer layer, thereby exposing phosphatidylserine and annexin-V at the external surface of the cell. Hence, this protein is suited to detect apoptotic cells. Because necrotic cells also expose phosphatidylserine as a result of loss of the membrane integrity, apoptotic cells must be differentiated from these necrotic cells with a concomitant use of a DNA dye, such as bisbenzimide, that allows the discrimination of necrotic cells from the apoptotic Annexin-V positive cells.

Cells from each condition were harvested and washed with PBS. Then they were incubated for 15 min with 100 μL of annexin-binding buffer (10mM HEPES, 140 mM NaCl, 2.5 mM CaCl2, pH 7.4) containing Annexin-V-Alexa Fluor® 568 antibody (1:50, Roche; 1:20, Life Technologies) and 5 mg/mL bisbenzimide. Finally, 20,000 cells for each condition were analyzed with the BD LSRFortessa SORP cytometer using the G610-A and V450-A lasers for the measurement of the Alexa Fluor® 568 dye (Annexin-V positive apoptotic cells) and bis-benzimide (late apoptotic/necrotic cells), respectively, in the Cytometry Service from the Germans Trias i Pujol Research Institute.

**Anchorage-independent growth analysis**

The anchorage-independent growing capacity of S462 cell line was assessed by performing the soft agar assay. In the siRNA transfection experiment, 24 h after of siRNA transfection, S462 cells from each condition were harvested and counted with the Countess^TM^ Automated Cell Counter (Life technologies). Then, a top agar layer was prepared by mixing the transfected cells with agarose and a total of 20,000 cells in a 0.8% agar layer were plated and allowed to solidify for each condition. Supplemented DMEM was added over the top agar layer and plates were incubated under standard conditions for two weeks, being medium replaced every week.

In the drug treatment experiments, a top agar layer was prepared by mixing untreated S462 cells with agarose, and a total of 20,000 cells in a 0.8% agar layer were plated and allowed to solidify for each condition. Supplemented DMEM containing the single or the combined drug was added over the top agar layer and plates were incubated under standard conditions for two weeks, being medium replaced every week.

***References***

1. Serra E. Schwann cells harbor the somatic *NF1* mutation in neurofibromas: evidence of two different Schwann cell subpopulations. *Hum Mol Genet*. 2000;9(20):3055-3064.

2. Terribas E, Garcia-Linares C, Lázaro C, Serra E. Probe-based quantitative PCR assay for detecting constitutional and somatic deletions in the *NF1* gene: Application to genetic testing and tumor analysis. *Clin Chem*. 2013;59(6):928-937.

3. Hellemans J, Mortier G, De Paepe A, Speleman F, Vandesompele J. qBase relative quantification framework and software for management and automated analysis of real-time quantitative PCR data. *Genome Biol*. 2008;8(2).

4. Pfaffl MW. A new mathematical model for relative quantification in real-time RT-PCR. *Nucleic Acids Res*. 2001;29(9):45e - 45.

5. Maertens O, Brems H, Vandesompele J, et al. Comprehensive *NF1* screening on cultured Schwann cells from neurofibromas. *Hum Mutat*. 2006;27(10):1030-1040.

6. Cong L, Ran FA, Cox D, et al. Multiplex genome engineering using CRISPR/Cas systems. *Science (80- )*. 2013;339(6121):819-823.

7. Kim H, Um E, Cho S-R, Jung C, Kim H, Kim J-S. Surrogate reporters for enrichment of cells with nuclease-induced mutations. *Nat Methods*. 2011;8(11):941-943.
